# Intraspecific Variation and Covariation of Functional Traits in *Phragmites australis* Across a Stagnant Constructed and a Dynamic Natural Wetland in Ganzhou, Jiangxi, China

**DOI:** 10.3390/plants15050692

**Published:** 2026-02-25

**Authors:** Mingyang Yu, Hong Zhu, Yuhui Wang, Wenlong Sun, Meiqi Yin, Yongda Chen, Lele Liu, Weihua Guo

**Affiliations:** 1 School of Life Sciences, Shandong University, 72 Binhai Road, Qingdao 266237, China; 2 National Glycoengineering Research Center, Shandong University, 72 Binhai Road, Qingdao 266237, China; 3 College of Chemistry, Chemical Engineering and Materials Science, Shandong Normal University, Jinan 250014, China; 4 School of Geography and Environmental Engineering, Gannan Normal University, Shida South Road, Ganzhou 341000, China

**Keywords:** common reed, functional traits, city wetlands, intraspecific variation

## Abstract

Urban wetlands, encompassing both natural and constructed ecosystems, are vital for urban resilience. Understanding how plant functional traits adapt to these distinct habitats is crucial for ecological management. This study investigates the intraspecific variation and trait covariation patterns of the common reed (*Phragmites australis*) in two contrasting urban wetland types in Ganzhou City: a stagnant, engineered constructed wetland and a dynamic, natural riverine wetland. This contrast represents a key gradient in hydrological regime and anthropogenic influence. We measured 22 morphological and chemical traits to assess trait differences, variability (coefficient of variation), and correlation patterns. Volcano plot analysis revealed significant habitat effects: reed in natural wetlands exhibited higher levels of Cu, P, N, and leaf moisture content (LMC), whereas those in constructed wetlands had higher Ca content. Traits such as Na, Mn, and Al showed high intraspecific variability. Correlation analyses revealed significant trade-offs and integrations among traits, such as positive correlations between LMC and nutrients (K, Cu), and negative correlations between Ca and key leaf morphological traits. Principal component analysis (PCA) further confirmed a significant separation along PC1, driven primarily by nutrient elements (Cu, P, K) and LMC, with natural wetlands scoring higher. In contrast, PC2, associated with leaf morphological traits (e.g., leaf area, leaf width), showed no significant inter-habitat difference. Our findings demonstrate that *P. australis* employs distinct ecological strategies by adjusting its functional traits and resource allocation in response to different urban wetland environments. This highlights the critical role of intraspecific trait variation in plant adaptation and has important implications for wetland restoration and the design of constructed ecosystems.

## 1. Introduction

Urban wetlands are critical yet often undervalued components of city landscapes [1]. These ecosystems include both natural features, such as rivers, and engineered ones, like constructed wetlands for landscape greening, water treatment, or both. They provide indispensable services that significantly enhance urban resilience and human well-being [2]. Wetland vegetation forms the structural and functional backbone of these aquatic ecosystems [3]. It is widely recognized that high species diversity enhances ecosystem stability and multifunctionality [4]. However, wetlands often tend toward plant community simplification due to the inherently stressful habitat conditions [5,6], including prominent monocultures of dominant species. Emerging evidence indicates that intraspecific variation can enhance niche differentiation and functional complementarity, thereby supporting key ecosystem functions like carbon sequestration [7,8]. Thus, in urban wetlands where species diversity is often constrained, intraspecific variability plays an essential role in sustaining ecological stability and service provision [9]. A deeper understanding of this dimension of diversity is critical for effective wetland conservation and restoration in anthropogenically altered environments.

Intraspecific variation refers fundamentally to differences in phenotype among individuals of the same species [10]. Functional traits are of particular interest because they directly influence an individual’s survival, development, growth, and reproduction [11]. These measurable traits reflect key physiological, morphological, and phenological attributes that not only determine plant fitness but also mediate ecosystem processes [12]. By analyzing functional traits, we gain valuable insight into how plant strategies and environmental performance are linked to ecological functions. This approach helps bridge the scale from individual adaptations to broader ecosystem services.

However, the various functional traits of an individual do not change in isolation, as each organism operates under a finite total resource budget including energy, nutrients, and time that cannot be allocated limitlessly across all physiological processes [13]. For instance, a high photosynthetic rate in plants is often associated with shorter leaf lifespan, as it requires high nitrogen content and low leaf mass per area, which increases the leaf’s vulnerability to herbivory and physical damage [14,15]. Consequently, individuals must distribute these resources among their functional traits to maximize their fitness, specifically survival and reproductive success. This strategic allocation of resources is referred to as an ecological strategy [16]. Moreover, what are central to this concept are the principles of trade-off and integration. Trade-off is defined as follows: due to resource limitation, investing more in one functional trait often necessitates reduced investment in another, resulting in a negative correlation between them [17,18]. Furthermore, natural selection does not optimize traits in isolation but acts on integrated suites of traits that work together coherently to execute a particular ecological strategy. Concepts like Grime’s CSR (Competitor, Stress-tolerator, Ruderal) theory provide frameworks for understanding how these trade-offs and integrations shape plant life histories and their distribution across environmental gradients, including the heterogeneous conditions found in urban wetlands [16,19].

*Phragmites australis* is a typical perennial emergent macrophyte that colonizes shallow-water wetland habitats, often forming zonal or extensive patchy distributions [20]. Owing to its vigorous clonal propagation and strong resource competitiveness, it readily establishes monodominant stands [20]. Furthermore, this species exhibits high phenotypic plasticity and considerable genetic diversity, enabling its traits to vary significantly in response to environmental conditions [17,21,22]. Therefore, analyzing its functional traits may provide key indicators for revealing the differential adaptive strategies employed by *P. australis* in both constructed and natural wetlands.

Hydrological disturbance gradients are fundamental drivers of plant community assembly and functional strategy expression in wetland ecosystems. Dynamic riverine systems are characterized by periodic flood pulses that create temporal windows of resource availability while imposing physical disturbance, theoretically favoring ruderal traits for rapid establishment and resource-acquisitive strategies during recovery phases [16,19]. In contrast, stagnant constructed wetlands experience persistent waterlogging and limited hydrological connectivity, imposing chronic abiotic stress (such as reduced oxygen availability, solute accumulation, and nutrient limitation) that should select for stress-tolerator traits, including structural reinforcement and conservative resource use [16]. These contrasting selective regimes provide a mechanistic framework for predicting how *P. australis* should adjust its functional traits across wetland types.

This study investigates the adaptive strategies of *P. australis* by comparing functional trait variations between two contrasting urban wetland types: a stagnant constructed reservoir and a dynamic natural river. We hypothesized that the distinct hydrological regimes (stagnant vs. flowing water) and associated anthropogenic management would select for divergent phenotypic syndromes: the natural wetland was expected to favor a resource-acquisitive strategy (reflected in higher nutrient content and growth-related traits), whereas the constructed wetland was expected to favor a stress-tolerant strategy (reflected in greater structural investment and conservative traits). To test this, we first examined habitat-based differences in mean trait values. Next, we quantified the extent of intraspecific trait variation within each habitat. Finally, we analyzed patterns of trait covariation to reveal the underlying trade-offs and ecological strategies. This research aims to provide mechanistic insights into plant adaptation in urban ecosystems, with implications for wetland restoration and landscape planning.

## 2. Results

### 2.1. Variation in Functional Trait and Environmental Properties Between Two Habitats

**Figure 1:**
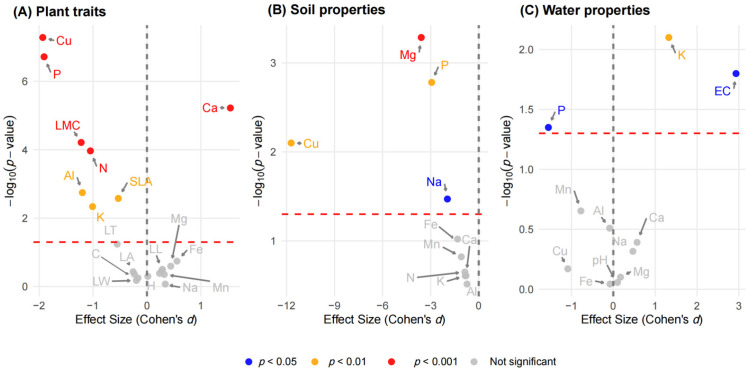
Volcano plots comparing differences in (**A**) plant functional traits of *Phragmites australis*, (**B**) soil properties, and (**C**) water properties between stagnant constructed and dynamic natural wetlands. Each point represents a single measured variable. The *x*-axis shows Cohen’s *d* effect size (positive: higher mean in constructed wetland; negative: higher mean in natural wetland). The *y*-axis shows −log_10_(*p*) from t-tests or Mann–Whitney tests, as appropriate. Horizontal dashed lines mark the significance threshold (*p* = 0.05). Point colors indicate significance levels: red (*p* < 0.001), orange (*p* < 0.01), blue (*p* < 0.05), and gray (*p* ≥ 0.05).

Utilizing established differential thresholds (*p* < 0.05 and |Cohen’s *d*| > 0.8), we determined that the natural wetland population exhibited significantly higher levels of Cu, P, N, and leaf moisture content (LMC) compared to the constructed wetland population, whereas Ca content was significantly higher in the constructed wetland population (Figure 1A).

Soils in the natural wetland exhibited significantly higher concentrations of Mg, P, Cu, and Na than those in the constructed wetland (all *p* < 0.05) (Figure 1B). No significant habitat differences were detected for the remaining measured elements (N, K, Mn, Fe, Al, Ca). The natural wetland had significantly higher water P concentrations (*p* < 0.05). Conversely, the constructed wetland showed significantly higher electrical conductivity (EC) and K concentrations (*p* < 0.05). Other water parameters (pH, Cu, Fe, Al, Mg, Mn, Na, Ca) did not differ significantly between the two sites (Table 1).

We found that in constructed wetlands, the traits exhibiting the greatest variability were sequentially Na, Mn, Al, and Cu. Carbon content was the least variable at only 3.6%, followed by the relatively stable traits: leaf length (LL), LMC, P, and leaf thickness (LT). Conversely, in natural wetlands, high variation was observed in Mn, SLA, Na, LDW, and Al, while C, N, P, and LMC were less variable (Table 2). Overall, Na, Al, Mn, and SLA demonstrated high variability; however, with the exception of SLA, most morphological traits and the C and N contents exhibited low variation across the study sites.

### 2.2. Trait Covariance Patterns in Two Habitats

**Figure 2:**
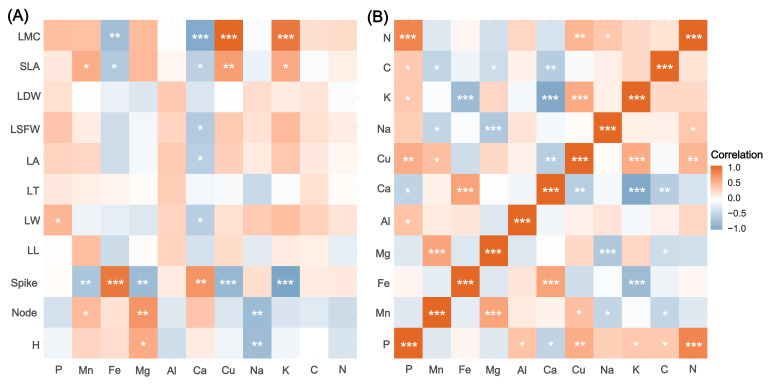
Spearman correlation heatmap of plant functional traits. (**A**) Spearman’s correlation matrix between plant morphological traits and elemental contents. (**B**) Correlation matrix among all measured elemental contents. Color intensity and hue represent the magnitude and sign of Spearman’s correlation coefficient, with red indicating positive correlations and blue indicating negative correlations. Asterisks indicate the statistical significance levels: *** *p* < 0.001, ** *p* < 0.01, * *p* < 0.05. Notes: H, height; Node, node number; Spike, spike number per shoot; LL, leaf length; LW, leaf width; LT, leaf thickness; LA, leaf average area; LSFW, leaf saturated fresh weight; LDW, leaf dry weight; SLA, specific leaf area; LMC, leaf moisture content; C, carbon content; N, nitrogen content; P, phosphorus content; Mn, manganese content; Fe, iron content; Mg, magnesium content; Al, aluminum content; Ca, calcium content; Cu, copper content; Na, sodium content; K, potassium content.

Spearman correlation coefficients were calculated to analyze the relationships and variations among the traits (Figure 2A). For morphological traits and mineral elements, LMC exhibited significantly positive correlations with P, Cu, and K, while showing a negative correlation with Fe and Ca. Spike number was positively correlated with Fe and Ca, but negatively correlated with Cu and K. Mg demonstrated positive correlations with both Node and H, yet a negative correlation with spike number. Furthermore, Ca was negatively correlated with LW, LA, and LSFW.

Regarding the relationships among the mineral elements (Figure 2B), P was positively correlated with N, K, Al, and Cu, but negatively correlated with Ca. Mn showed a negative correlation with C and Na, and a positive correlation with Mg. Fe was negatively correlated with K and Cu, whereas it exhibited a positive correlation with Ca. Mg was also negatively correlated with Na and C. Notably, Ca showed extensive negative correlations with N, C, K, and Cu. Conversely, Cu maintained a stable positive correlation with both K and N.

### 2.3. PCA

**Figure 3:**
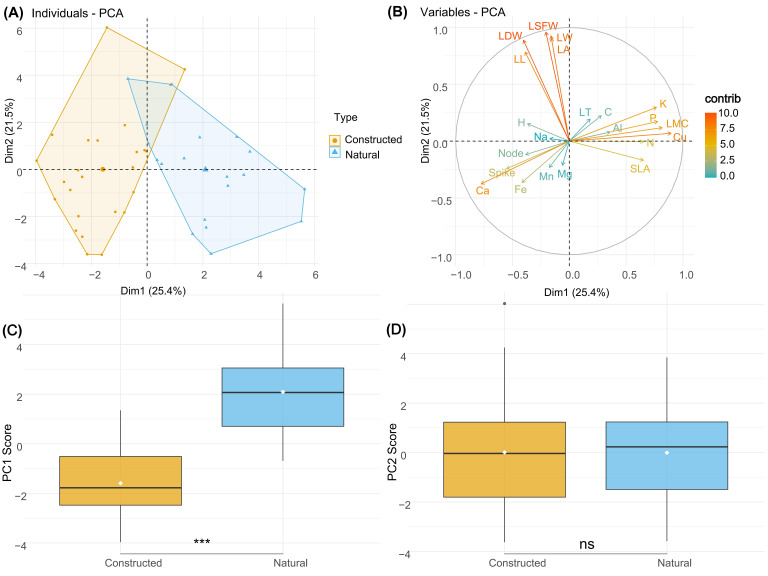
Principal component analysis (PCA) of plant traits from constructed and natural wetlands. (**A**) PCA biplot showing individual samples colored by wetland type with convex hulls. (**B**) Variable contribution plot displaying the loading of each trait on the first two principal components. (**C**) Comparison of PC1 scores between constructed and natural wetlands. (**D**) Comparison of PC2 scores between constructed and natural wetlands. Asterisks indicate statistical significance levels: *** *p* < 0.001; ns, not significant. In picture C and D, the white dots represent the mean value of each group. Notes: H, height; Node, node number; Spike, spike number per shoot; LL, leaf length; LW, leaf width; LT, leaf thickness; LA, leaf average area; LSFW, leaf saturated fresh weight; LDW, leaf dry weight; SLA, specific leaf area; LMC, leaf moisture content (dry mass basis); C, carbon content; N, nitrogen content; P, phosphorus content; K, potassium content; Mn, manganese content; Fe, iron content; Mg, magnesium content; Al, aluminum content; Ca, calcium content; Cu, copper content; Na, sodium content.

Principal component analysis (PCA) revealed distinct patterns in plant trait variation between constructed and natural wetlands. The proportion of each trait in the first five principal components was calculated (Table S3). The first two principal components collectively explained 45.1% of the total variance in the dataset (Figure 3A). PC1, which accounted for 27.6% of the variance, showed significant differentiation between wetland types (*p* < 0.05) (Figure 3C). Natural wetlands exhibited significantly higher scores along PC1 compared to constructed wetlands. The traits contributing most substantially to PC1 included Cu, Ca, P, K, and LMC (Figure 3B), indicating that chemical element concentrations and leaf structural traits were primary drivers of separation between wetland types along this axis. In contrast, PC2, explaining 17.5% of the variance, did not show significant differences between constructed and natural wetlands (*p* > 0.05) (Figure 3D). The major contributors to PC2 were leaf-related morphological traits, including LSFW, LW, LA, LDW, and LL (Figure 3B).

The PERMANOVA confirmed a statistically significant effect of habitat type on the overall multivariate trait composition (pseudo-*F* = 7.87, *p* < 0.001), with habitat explaining approximately 15.8% (*R*^2^ = 0.158) of the total trait variation. The test for homogeneity of multivariate dispersions was not significant (*p* = 0.309), validating the PERMANOVA model assumption. This result provides statistical support for the habitat differentiation visually observed in the PCA ordination.

### 2.4. CSR Strategy Analysis

**Figure 4:**
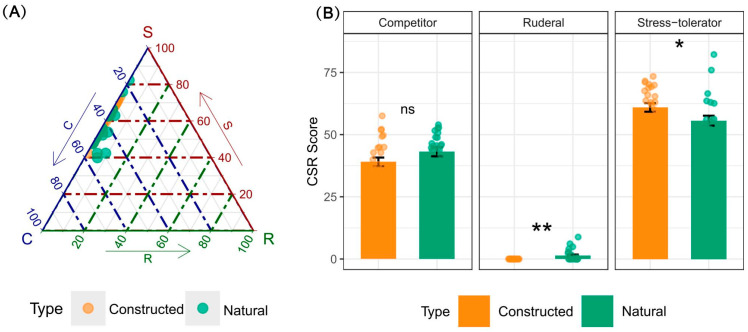
CSR strategy analysis of *Phragmites australis* in constructed and natural wetlands. (**A**) Ternary plot illustrating the distribution of individual plants along the axes of competitor (C), stress-tolerator (S), and ruderal (R) strategies. Each point represents a single ramet, colored by habitat type. (**B**) Bar charts showing the mean (± SE) score for each CSR component in the two habitats. Individual data points (jittered) are overlaid. Asterisks above the bars indicate the level of statistical significance from one-way ANOVAs (* *p* < 0.05, ** *p* < 0.01; ns, not significant).

The CSR strategy analysis revealed distinct functional profiles for *P. australis* between the two wetlands (Figure 4). While competitor (C) scores did not differ significantly between habitats (Constructed: 39.0 ± 1.7; Natural: 43.1 ± 1.8; *p* = 0.114), the populations diverged along the stress-tolerator (S) and ruderal (R) axes. Plants in the constructed wetland exhibited significantly higher stress-tolerator scores (61.0 ± 1.7 vs. 55.6 ± 2.0 in the natural wetland; *p* < 0.05). Conversely, the natural wetland population showed a significantly higher ruderal score (1.3 ± 0.5 vs. 0.0 ± 0.0 in the constructed wetland; *p* < 0.01).

## 3. Discussion

The pronounced trait divergence in *P. australis* between the natural and constructed wetlands highlights the powerful role of habitat as a filter shaping functional phenotypes. The natural wetland, with its presumably dynamic hydrology and complex nutrient dynamics, fostered a resource-acquisitive strategy [23]. This was manifested in significantly higher foliar concentrations of N, P, and K, elements central to photosynthetic machinery, energy transfer, and osmotic regulation, thereby facilitating rapid growth and high productivity [24]. Conversely, the engineered environment of the constructed wetland favors a stress-tolerant strategy due to its engineered conditions, exemplified by a marked accumulation of Ca for enhanced structural support and cellular integrity [25,26].

Beyond the primary macronutrients, the differential accumulation of specific elements further elucidates the contrasting physiological states [27,28]. The elevated Cu content in natural wetland plants likely reflects its vital role as a cofactor in enzymes critical for respiration and antioxidant defense, pointing towards a more metabolically active state [29]. Conversely, the high Ca content in constructed wetland reeds is not merely a passive uptake but an active investment in cell wall fortification and membrane stability, a fundamental adaptation to mitigate physical and ionic stresses prevalent in human-made environments [30]. This strategic allocation is further evidenced by the significantly higher LMC in the natural habitat, supporting turgor-driven growth, whereas the constructed wetland plants exhibited a different set of priorities [19].

The two wetlands differ fundamentally in hydrological regime: the constructed wetland is a stagnant, impounded reservoir, whereas the natural wetland is a dynamic, flow-through riverine system [31]. This contrast is reflected in our abiotic measurements. The natural wetland exhibited significantly higher water P concentrations, likely sustained by fluvial inputs and sediment–water exchange, alongside elevated soil P, Mg, Cu, and Na, consistent with alluvial nutrient enrichment. In contrast, the constructed wetland showed higher water EC and K concentrations, possibly resulting from evaporative concentration, slow water turnover, or the weathering of construction materials [25]. These environmental filters appear to have shaped distinct adaptive syndromes in *P. australis* through the physiological mechanisms detailed below. The nutrient-rich, well-oxygenated conditions of the natural wetland align with the resource-acquisitive strategy observed there (high foliar N, P, K, Cu; high LMC), which supports rapid biomass production by fueling photosynthetic metabolism and maintaining cell turgor for expansion growth [32]. Conversely, the combination of elevated EC and lower P availability in the constructed wetland may impose osmotic and nutritional stress, triggering active stress-tolerance responses characterized by structural reinforcement (high foliar Ca) and reduced leaf expansion [30]. These results suggest that the primary environmental filters driving trait divergence are hydrological connectivity and its cascading effects on nutrient and ion dynamics. These findings underscore the value of integrating direct abiotic measurements into trait-based studies of urban wetlands.

The observed trait shifts are mechanistically grounded in the contrasting hydrological regimes. In the dynamic natural wetland, periodic flooding and drawdown sustain aerobic conditions that facilitate rapid nutrient uptake and metabolic activity. Elevated foliar N, P, and Cu directly support photosynthetic machinery and antioxidant defense, while high LMC maintains turgor for cell expansion during post-flood recovery [29,32]. Conversely, in the stagnant constructed wetland, persistent waterlogging imposes hypoxia and ionic stress (elevated EC), limiting root function and nutrient availability. Under these constraints, plants actively accumulate Ca to reinforce cell walls and stabilize membranes, a key stress-tolerance mechanism [26,30]. This structural investment, however, incurs a direct cost: the negative Ca–leaf morphology correlation reflects a trade-off between stress defense and expansive growth, consistent with growth–defense theory [33,34]. Thus, the trait divergence represents coordinated physiological adjustments to distinct environmental filters, rather than passive elemental accumulation.

The CSR scores quantitatively capture how these trait syndromes reflect adaptive trade-offs under contrasting flood regimes [16,19]. These individual trait responses did not occur in isolation but were structured through patterns of trait covariation, revealing underlying trade-offs [18]. Although the first two PCA axes captured the dominant dimensions of this trait covariation (45.1% of total variance), our interpretation based on them represents a simplified view of a more complex, multidimensional trait structure in *P. australis*. The strong positive correlations among N, P, K, and Cu formed an integrated module for efficient resource acquisition and utilization [33,34,35]. Critically, the negative correlation between Ca and key leaf morphological traits like LW and LA revealed a core trade-off: investment in structural reinforcement and stress tolerance via Ca came at the direct cost of reducing leaf expansion and light interception capacity, a hallmark of a growth–defense balance [36,37,38]. The integration of these trait patterns is consistent with the conceptual framework of CSR theory and was quantitatively reflected in the CSR scores (Figure 4). The natural wetland populations, with their high nutrient content and associated traits, reflected a trait syndrome aligned with a competitor strategy (C-selected), optimized for resource capture, yet were also characterized by a significant ruderal (R) component, suggesting a mixed C-R strategy in a dynamic environment [19]. The constructed wetland populations, characterized by structural investment (high Ca) and a concomitant reduction in expansive growth, demonstrate a clear shift towards a stress-tolerator strategy (S-selected) as their dominant adaptive response, prioritizing survival and persistence in a more challenging and potentially resource-limited setting.

Our assessment of intraspecific trait variation is based on the operational assumption that sampled ramets spaced ≥100 m represent distinct genetic individuals. Given the extensive clonal spread possible in *P. australis* [39], it is possible that some of the measured variation reflects within-genet phenotypic plasticity rather than (or in addition to) genetic differentiation. This implies that our study likely captures phenotypic variation at the habitat scale, which is ecologically relevant as it reflects the actual functional traits expressed in each environment [40]. Our estimates of genetically based intraspecific trait variation might be conservative if plasticity homogenizes traits within clones across habitats. Conversely, the observed significant trait divergence between wetlands suggests strong environmental filtering, capable of generating patterns even among potentially connected ramets. Future studies employing genetic markers and common garden experiments [22,27] would be valuable to precisely partition the observed intraspecific trait variation into its genetic and plastic components, further clarifying the adaptive significance of the documented trait syndromes. In addition, our study captured trait expression at a single time point (peak growing season). While this reveals distinct and coherent habitat-specific syndromes, future research incorporating seasonal monitoring would be valuable to disentangle the dynamics of phenotypic plasticity and stable adaptation in these systems.

These findings carry significant implications for wetland conservation and construction. They demonstrate that successful ecosystem establishment depends not only on species presence but also on the development of appropriate functional trait syndromes. For constructed wetlands designed for water purification, management should aim to promote the nutrient-rich, acquisitive strategy to enhance bioremediation. Conversely, in habitats requiring stability under physical stress, the stress-tolerant strategy may be more desirable. Therefore, restoration ecology must adopt a trait-based perspective, consciously managing environmental filters to steer plant populations toward the functional profiles that ensure both ecosystem resilience and the targeted provision of ecosystem services.

## 4. Materials and Methods

### 4.1. Study Region and Sampling Design

**Figure 5:**
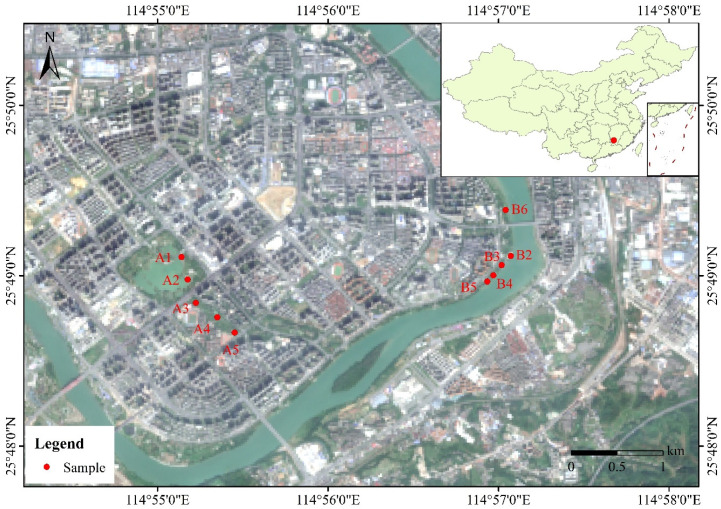
The sampling locations of *Phragmites australis* populations in Ganzhou city, Jiangxi province, China. In our study, the areas A1 to A5 represent sampling points for reed populations along the Central Park waterbody (i.e., artificial wetland), while the areas B2 to B6 represent sampling points for reed populations along the Zhangjiang River (i.e., natural wetland).

The study was conducted in Ganzhou City (24°29′ N–27°09′ N, 113°54′ E–116°38′ E), located in the southern part of Jiangxi Province, China in August of 2025. This region experiences a humid subtropical monsoon climate characterized by distinct seasons, abundant rainfall, and a mean annual temperature. The primary river system is the Zhangshui River (a major tributary of the Ganjiang River, itself part of the Yangtze River basin), shaping much of the local topography. Dominant vegetation includes subtropical evergreen broad-leaved forests, with significant areas of coniferous plantations and secondary shrublands, alongside riparian vegetation corridors [41]. Two kinds of contrasting wetlands were selected as sampling sites for this survey: a natural riparian wetland along the Zhangshui River and an artificially constructed wetland in the Central Park. Five stands (a stand means a continuous reed land, where the reeds are in the same height, age, and conditions) of *P. australis* were selected in each wetland (Figure 5), and 5 shoots were selected from each stand. Within each wetland, sampling stands were spaced at least 100 m apart. This distance was chosen to minimize the probability of sampling ramets (stems/shoots) belonging to the same genetic individual (genet), given the clonal growth habit of *P. australis*. Within each stand, five ramets were sampled, each separated by more than 1.5 m to further avoid collecting from the same immediate clone. We acknowledge that without genetic confirmation, the genetic independence of all sampled units cannot be definitively verified. This sampling protocol is, however, a practical assumption in field-based trait studies of *P. australis* when assessing population- or habitat-level phenotypic variation [39,40].

### 4.2. Functional Traits Measurements

Standardized growth measurements were recorded for each tagged individual (shoot). Shoot height (cm) was measured from the base of the main stem at soil level to the apex of the longest shoot or reed inflorescence using a measuring tape. Shoot diameter (mm), representing stem thickness, was measured at 10 cm height above the base using a digital vernier caliper. The total number of nodes (points of leaf attachment) present on the main stem was counted. For each shoot, the spike status was recorded as the presence (1) or absence (0) of a spike/inflorescence during the survey period. This binary measurement per shoot was then used in subsequent analyses. All measurements were taken by the same observer to minimize bias.

Furthermore, we collected three healthy, green, and fully shaped leaves from each individual as close to the tip as possible for leaf trait analysis. Morphological traits included leaf length (LL, mm) and leaf width (LW, mm), measured using a ruler, leaf thickness (LT, mm) used the digital vernier caliper, leaf area (LA, mm), saturated fresh weight (SFW, g) and dry weight (DW, g). LA was quantified using CamScanner software (v.6.76; Shanghai Hehe Technology Corporation Limited, Shanghai, China) and analyzed with ImageJ software (v.1.51; National Institutes of Health, Bethesda, MD, USA). After soaking in purified water overnight to achieve full hydration and blotting dry, the saturated fresh weight (SFW) was measured. The same samples were then oven-dried (105 °C for 30 min, then 80 °C for 24 h) to constant mass to determine the dry weight (DW) using a precision balance (±0.0001 g). Based on these measurements, leaf moisture content (LMC) was calculated as (SFW − DW)/DW × 100% and expressed on a dry mass basis. Meanwhile, 11 chemistry elements conductions are done. The 9 elements (P, Mn, Fe, Mg, Al, Ca, Cu, Na, K) were determined using inductively coupled plasma optical emission spectrometry (ICP-OES; iCAP Pro, Thermo Scientific, Bremen, Germany) (GB 5009.268-2016), while nitrogen content was measured using a fully automated Kjeldahl nitrogen analyzer (Shanghai INESA Scientific Instrument Co., Ltd., Shanghai, China) (NYT 2017-2011). Carbon content was determined using an elemental analyzer (Thermo Scientific FLASH 2000, Waltham, MA, USA).

### 4.3. Environmental Factors Measurements

To characterize the abiotic environment of each wetland, we collected soil and water samples from both the constructed and natural riparian sites. At each site, five composite soil samples were obtained by randomly collecting and thoroughly mixing subsurface soil (0–10 cm depth, after removing the litter layer) from five stands. Five replicate water samples were collected from multiple points within each wetland. Soil samples were analyzed for pH (PHSJ-3F pH meter, Shanghai INESA Scientific Instrument Co., Ltd., Shanghai, China), electrical conductivity (EC; DDSJ-318T conductivity meter, Shanghai INESA Scientific Instrument Co., Ltd., Shanghai, China), organic carbon, and total nitrogen (elemental analyzer; FLASHSMART, Thermo Scientific, Milan, Italy), as well as total concentrations of P, K, Mn, Fe, Mg, Al, Ca, Cu, and Na. Water samples were analyzed for pH, EC, and concentrations of Cu, Fe, Al, K, Mg, Mn, Na, Ca, and P. All metal elements (P, K, Mn, Fe, Mg, Al, Ca, Cu, Na) in both soil and water were determined using inductively coupled plasma optical emission spectrometry (ICP-OES).

### 4.4. Data Analysis

The variability of each plant trait was assessed by calculating the coefficient of variation (CV) for constructed sites, natural sites, and the entire dataset, with results expressed as percentages. Means and standard errors were computed for each group after excluding missing values. Differences between site types were evaluated using a dual statistical approach: independent samples t-tests for traits meeting assumptions of normality (Shapiro–Wilk test) and homogeneity of variance (Levene’s test), and Mann–Whitney U tests for traits violating these assumptions. The magnitude of differences was quantified using Cohen’s *d* effect sizes, and results were visualized through a volcano plot displaying effect sizes against statistical significance (−log_(10)_ *p*-values), with significance thresholds and trait annotations. To identify traits that exhibited not only statistically significant but also ecologically meaningful differences between habitats, statistical significance was assessed at *p* < 0.05. Following conventional criteria, an absolute value of Cohen’s *d* > 0.8 was considered indicative of a “large” effect size, a threshold that, in this system, corresponds to biologically meaningful trait differences associated with shifts between acquisitive and conservative strategies in wetland plants [42]. In our analysis, applying this stringent dual criterion reclassified only the SLA trait compared to using *p* < 0.05 alone, indicating that the overall pattern of trait differentiation was robust. For soil and water properties, which serve to characterize the environmental context rather than to assess biological effect sizes, we considered only statistical significance (*p* < 0.05) without an effect size threshold.

Spearman correlation analyses were conducted to examine relationships between non-elemental (morphological) and elemental traits. Additionally, autocorrelation analysis among elemental traits was performed to identify patterns of nutrient associations. Both correlation matrices were visualized through hierarchical clustering heatmaps.

Principal Component Analysis (PCA) was performed on standardized trait data to identify major patterns of variation. Results were visualized through factor maps showing individual scores grouped by site type with convex hulls, and variable contributions represented by vectors. Differences between site types along the first two principal components were assessed using independent samples t-tests and displayed with significance annotations.

To statistically test for multivariate differences in functional traits between the two wetland habitats, a Permutational Multivariate Analysis of Variance (PERMANOVA) was performed using the adonis2 function in the vegan R package (v2.6–10) [43]. The analysis was based on a Euclidean distance matrix derived from the standardized trait data, with habitat type as the fixed factor. Significance was assessed with 9999 permutations. The assumption of homogeneity of multivariate group dispersions was tested using the betadisper function with the same number of permutations.

To quantify the ecological strategies of *P. australis*, we calculated competitor (C), stress-tolerator (S), and ruderal (R) scores based on three key functional traits: leaf area (LA, mm^2^), leaf dry matter content (LDMC, mg·g^−1^), and specific leaf area (SLA, mm^2^ mg^−1^) [16]. The calculation was performed using the CSR function in the MultiTraits R package (v0.6.0) [44]. Differences in C, S, and R scores between the constructed and natural wetlands were assessed using separate one-way analyses of variance.

All analyses were conducted in R v4.5.2 software.

## 5. Conclusions

Our study demonstrates that *P. australis* exhibits significant trait differentiation between natural and constructed wetlands. The data directly show that individuals in natural wetlands invest more in nutrients (N, P, K, Cu) and leaf moisture content, while those in constructed wetlands accumulate higher Ca levels. Trait correlation analyses reveal consistent trade-offs (e.g., negative Ca-leaf morphology relationships) and synergies (e.g., positive N-P-K-Cu associations) that are statistically robust and ecologically interpretable. CSR analysis further quantifies these patterns, showing that natural wetland populations exhibit a mixed C-R strategy, while those in constructed wetlands are clearly S-selected. These empirically supported patterns are consistent with, though not definitive proof of, adaptive interpretations based on resource-acquisition versus stress-tolerance frameworks. The inclusion of environmental data allows us to link observed trait variation to specific habitat filters (hydrological regime, nutrient availability, and ionic stress), providing mechanistic context. However, because our study is observational and captures a single time point, we cannot fully distinguish genetically based adaptation from phenotypic plasticity. Future common garden experiments and genetic analyses would be necessary to partition these components and confirm evolutionary adaptation. Nevertheless, our findings demonstrate that intraspecific trait variation and coordinated trait syndromes are key mechanisms enabling *P. australis* to persist across heterogeneous urban wetland habitats, with important implications for the ecological restoration and design of constructed wetlands.

## Figures and Tables

**Table 1:** Functional traits (mean ± se) of *Phragmites australis* from constructed and natural wetland in Ganzhou city, China.

Trait	Constructed	Natural	Overall
H (cm)	217.36 ± 10.49	203.96 ± 12.32	210.66 ± 8.06
Node	20.64 ± 0.98	19.20 ± 1.02	19.92 ± 0.71
Spike	0.32 ± 0.10	0.04 ± 0.04	0.18 ± 0.05
LL (cm)	42.33 ± 1.53	39.64 ± 2.33	40.98 ± 1.39
LW (cm)	2.34 ± 0.11	2.48 ± 0.17	2.41 ± 0.10
LT (mm)	0.19 ± 0.01	0.22 ± 0.01	0.21 ± 0.01
LA (mm^2^)	3293.72 ± 298.21	3720.18 ± 362.09	3506.95 ± 234.12
LSFW (g)	3.71 ± 0.32	4.04 ± 0.47	3.87 ± 0.28
LDW (g)	1.40 ± 0.10	1.39 ± 0.18	1.39 ± 0.10
SLA (mm^2^·mg^−1^)	7.10 ± 0.30	9.86 ± 1.44	8.48 ± 0.75
LMC (%)	161.2 ± 6.0	205.0 ± 8.2	183.1 ± 5.9
C (%)	47.57 ± 0.36	47.95 ± 0.33	47.76± 0.24
N (%)	2.34 ± 0.13	2.97 ± 0.12	2.66 ± 0.10
P (%)	0.15 ± 0.01	0.22 ± 0.01	0.18 ± 0.01
K (%)	1.39 ± 0.08	1.87 ± 0.12	1.63 ±0.08
Mn (mg·kg^−1^)	171.68 ± 30.16	131.77 ± 20.83	151.73 ± 18.37
Fe (mg·kg^−1^)	80.72 ± 7.32	65.05 ± 3.94	72.88 ± 4.27
Mg (mg·kg^−1^)	1156.18 ± 89.02	1005.07 ± 47.94	1080.63 ± 51.24
Al (mg·kg^−1^)	323.28 ± 56.90	810.61 ± 105.40	572.36 ± 70.31
Ca (mg·kg^−1^)	4024.96 ± 322.55	1947.42 ± 230.24	2986.19 ± 249.74
Cu (mg·kg^−1^)	3.03 ± 0.42	6.89 ± 0.41	4.96 ± 0.41
Na (mg·kg^−1^)	63.20 ± 13.51	45.85 ± 6.67	54.52 ± 7.56

Notes: H, height; Node, node number; Spike, spike number per shoot; LL, leaf length; LW, leaf width; LT, leaf thickness; LA, leaf average area; LSFW, leaf saturated fresh weight; LDW, leaf dry weight; SLA, specific leaf area; LMC, leaf moisture content (dry mass basis); C, carbon content; N, nitrogen content; P, phosphorus content; K, potassium content; Mn, manganese content; Fe, iron content; Mg, magnesium content; Al, aluminum content; Ca, calcium content; Cu, copper content; Na, sodium content.

**Table 2:** Coefficient of variation (CV) of *Phragmites australis* from constructed and natural wetland in Ganzhou city, China.

Trait	Constructed CV	Natural CV	Overall CV
H	24.1%	30.2%	27.1%
Node	23.8%	26.5%	25.1%
Spike	148.8%	500.0%	215.6%
LL	18.0%	29.4%	24.0%
LW	23.0%	34.6%	29.5%
LT	19.6%	23.2%	22.3%
LA	45.3%	48.7%	47.2%
LSFW	42.9%	57.7%	51.1%
LDW	36.6%	65.9%	52.7%
SLA	21.0%	72.8%	62.7%
LMC	18.7%	19.9%	22.8%
C	3.6%	3.3%	3.4%
N	26.4%	18.9%	25.1%
P	19.2%	22.6%	30.5%
K	26.0%	30.1%	32.3%
Mn	84.3%	75.8%	82.1%
Fe	43.5%	29.0%	39.7%
Mg	36.9%	22.9%	32.2%
Al	82.6%	62.4%	82.4%
Ca	38.4%	56.7%	56.7%
Cu	66.9%	28.6%	56.0%
Na	102.5%	69.7%	94.0%

Notes: H, height; Node, node number; Spike, spike number per shoot; LL, leaf length; LW, leaf width; LT, leaf thickness; LA, leaf average area; LSFW, leaf saturated fresh weight; LDW, leaf dry weight; SLA, specific leaf area; LMC, leaf moisture content (dry mass basis); C, carbon content; N, nitrogen content; P, phosphorus content; K, potassium content; Mn, manganese content; Fe, iron content; Mg, magnesium content; Al, aluminum content; Ca, calcium content; Cu, copper content; Na, sodium content.

## Data Availability

All data supporting the findings of this study are included as a Supplementary Information File with this manuscript.

## Supplementary Materials

The following supporting information can be downloaded at https://www.mdpi.com/article/10.3390/plants15050692/s1, Table S1: Raw trait measurements for individual *Phragmites australis* samples from constructed (A1–A5) and natural (B2–B6) wetland sites, Table S2: Statistical comparison of functional traits between constructed and natural wetland groups, Table S3: Principal Component Analysis (PCA) rotation/loading values for each trait across the first five dimensions (Dim.1–Dim.5).
